# Supplementary material for: The genome sequence of a sawfly, Athalia cordata Serville, 1823
Source: Wellcome Open Res. 2025 Jan 15;10:15. [Version 1] doi: 10.12688/wellcomeopenres.23456.1 (PMC11842964; doi:10.12688/wellcomeopenres.23456.1)
Supplement: Peer review history

# Reviewer report: Reviewer response for version 1

**DOI:** 10.21956/wellcomeopenres.25866.r118075

**Published:** 2025-02-20

**Authors:** Cheng Sun

**Affiliations:** 1 Chinese Academy of Agricultural Sciences, Beijing, China

*Athalia cordata* is a yellow and black species of sawfly. The authors produced Pacific HiFi and Hi-C reads for a female of this species and constructed a chromosome-level of reference genome. It seemed that the protein-coding genes of this genome were well assembled. I am wondering the repeat content of this genome. It will be interesting to investigate its repeat content as its genome size is relatively small (169 Mb).There should be a lack of TEs?

Are sufficient details of methods and materials provided to allow replication by others?

Yes

Is the rationale for creating the dataset(s) clearly described?

Yes

Are the datasets clearly presented in a useable and accessible format?

Yes

Are the protocols appropriate and is the work technically sound?

Yes

Reviewer Expertise:

insect genome evolution

I confirm that I have read this submission and believe that I have an appropriate level of expertise to confirm that it is of an acceptable scientific standard.

# Reviewer report: Reviewer response for version 1

**DOI:** 10.21956/wellcomeopenres.25866.r117461

**Published:** 2025-01-27

**Authors:** Jerome H L Hui

**Affiliations:** 1 The Chinese University of Hong Kong, Hong Kong SAR, Hong Kong

In this data note, Halstead and Falk obtained the genomic resource of a female hymenopteran sawfly *Athalia cordata*. In the literature, it seems that there are reports using *A. cordata *(Serville, 1823), while some other reports use *A. cordata *(Audinet-Serville, 1823). I had also tried but could not find the original article describing/naming this species; and if it's possible, the authors should also consider resolving this issue to ensure it is correctly stated. Prior to this report, the molecular data available for this species are limited to mitochondrial cytochrome oxidase subunit 1 (COI) sequences deposited to the NCBI database. Therefore, this new genomic resource will be useful for further studies, such as understanding their biology, ecology, population structure, response to environmental/climate changes, and evolutionary relationships with other insects.

 This genome resource is excellent from the summary statistics, with high BUSCO number scores, high sequence continuity (scaffold N50), and majority of sequences contained on the 4 pseudochromosomes (plus mitochondrion). To sum up, this is another valuable contribution by the Darwin Tree of Life Consortium.

Are sufficient details of methods and materials provided to allow replication by others?

Yes

Is the rationale for creating the dataset(s) clearly described?

Yes

Are the datasets clearly presented in a useable and accessible format?

Yes

Are the protocols appropriate and is the work technically sound?

Yes

Reviewer Expertise:

Genomics, evolution, invertebrates

I confirm that I have read this submission and believe that I have an appropriate level of expertise to confirm that it is of an acceptable scientific standard.
